# Clinic and Home Blood Pressure Lowering Effect of an Angiotensin Receptor Blocker, Fimasartan, in Postmenopausal Women with Hypertension

**DOI:** 10.1097/MD.0000000000003764

**Published:** 2016-06-03

**Authors:** Song-Yi Kim, Seung-Jae Joo, Mi-Seung Shin, Changsoo Kim, Eun Joo Cho, Ki-Chul Sung, Seok-Min Kang, Dong-Soo Kim, Seung Hwan Lee, Kyung-Kuk Hwang, Jeong Bae Park

**Affiliations:** From the Department of Internal Medicine (S-YK, S-JJ), Jeju National University School of Medicine, Jeju; Division of Cardiology (M-SS), Department of Internal Medicine, Gachon University Gil Medical Center, Incheon; Department of Preventive Medicine (CK), Yonsei University College of Medicine; Division of Cardiology (EJC), Department of Internal Medicine, St. Paul's Hospital, Catholic University of Korea; Division of Cardiology (K-CS), Department of Internal Medicine, Kangbuk Samsung Hospital, Sungkyunkwan University School of Medicine; Cardiology Division (S-MK), Severance Cardiovascular Hospital and Cardiovascular Research Institute, Yonsei University College of Medicine, Seoul; Division of Cardiology (D-SK), Paik Hospital, Inje University College of Medicine, Busan; Division of Cardiology (SHL), Wonju Severance Christian Hospital, Wonju Medical College, Yonsei University, Wonju; Department of Internal Medicine (K-KH), Chungbuk National University College of Medicine, Cheongju; and Division of Cardiology (JBP), Department of Internal Medicine, Cheil General Hospital, Dankook University College of Medicine, Seoul, South Korea.

## Abstract

Angiotensin receptor blockers may be an appropriate first-line agent for postmenopausal women with hypertension because the activation of renin–angiotensin–aldosterone system is suggested as one possible mechanism of postmenopausal hypertension. However, there are few studies substantiating this effect. This study aimed to investigate clinic and home blood pressure (BP) lowering effect of fimasartan, a new angiotensin receptor blocker, in postmenopausal women with hypertension.

Among patients with hypertension enrolled in K-Mets Study, 1373 women with fimasartan as a first antihypertensive drug and 3-months follow-up data were selected. They were divided into 2 groups; premenopausal women (pre-MPW; n = 382, 45.3 ± 4.6 years) and postmenopausal women (post-MPW; n = 991, 60.9 ± 8.2 years).

Baseline clinic systolic BP was not different (pre-MPW; 152.9 ± 15.2 vs. post-MPW; 152.8 ± 13.5 mm Hg), but diastolic BP was lower in post-MPW (pre-MPW; 95.7 ± 9.4 vs. post-MPW; 91.9 ± 9.4 mm Hg, *P* <0.001). After 3-month treatment, clinic BP declined effectively without significant differences between 2 groups (Δsystolic/diastolic BP: pre-MPW; −25.7 ± 17.7/−14.2 ± 11.3 vs. post-MPW; −25.7 ± 16.3/−13.1 ± 10.9 mm Hg). Home morning and evening systolic BP decreased similarly in both groups (Δmorning/evening systolic BP: pre-MPW; −21.3 ± 17.9/−23.1 ± 15.8 vs. post-MPW; −20.4 ± 17.3/−20.2 ± 19.2 mm Hg). Fimasartan also significantly decreased the standard deviations of home morning and evening systolic BP of pre-MPW and post-MPW.

Fimasartan was a similarly effective BP lowering agent in both post-MPW and pre-MPW with hypertension, and it also decreased day-to-day BP variability.

## INTRODUCTION

Aging is an important determinant of hypertension,^1–5^ but the age-related increase of systolic blood pressure (BP) is more rapid in women than in men after the mid-forties.^1,5^ The prevalence of hypertension in premenopausal women is much lower than that in men of similar age, but it increased abruptly after menopause.^1,4–11^ Other cardiovascular (CV) risk factors such as dyslipidemia, diabetes mellitus, or obesity also become to be more common in postmenopausal than in premenopausal women.^4,5,11,12^ Strict BP control with antihypertensive drug as well as lifestyle modification is recommended to minimize CV morbidity and mortality of postmenopausal women with hypertension.^12^

The renin–angiotensin–aldosterone system is a major body system for controlling BP and its blockers have been widely used in patients with hypertension to lower high BP.^13,14^ The renin–angiotensin–aldosterone system has been suggested to play an important role in postmenopausal hypertension because of its activation after menopause.^15–17^ Angiotensin-converting enzyme inhibitors (ACEIs) or angiotensin receptor blockers (ARBs) may be appropriate as a first-line agent for postmenopausal hypertension,^12^ and small studies showed the good antihypertensive effect of ACEIs or ARBs in postmenopausal women with hypertension.^18,19^ However, their BP-lowering effect in a large population of postmenopausal women with hypertension has rarely been determined, especially using measurements of home BP.

Home BP measurements by appropriately trained patients may be more reliable and reproducible than traditional clinic BP data, and it has been demonstrated that home BP was superior to clinic BP, and similar to ambulatory BP for predicting CV events.^20^ Recent guidelines for the management of hypertension recommended home BP measurements to accurately diagnose hypertension and assess the response to antihypertensive agents.^20,21^ Home BP measurements also provide day-to-day BP variability (BPV), which has been shown to be associated with stroke mortality,^22^ CV events,^23^ or hypertensive target organ damage.^24,25^

Fimasartan, a new ARB, has been demonstrated to effectively and safely control hypertension in the Safe-KanArb study.^26^ The objective of the present study was to investigate antihypertensive effect of fimasartan in the clinic and at home after 3-month treatment in postmenopausal women with low-to-moderate risk hypertension.

## METHODS

This investigation is a substudy of K-Mets Study, which is a prospective, multicenter, single-arm, observational study. The study design, demographic characteristics, baseline BP data, and metabolic risk factors have been already described.^27^ This study was approved by the institutional review board of Cheil General Hospital on behalf of 582 primary care clinics. Another 10 university hospitals approved this study through their own institutional review board. All study subjects signed the informed consents.

### Study Population

Between October 17, 2011 and October 31, 2012, total 10,601 hypertensive patients were enrolled from 582 primary care clinics and 11 university hospitals in South Korea. Patients with hypertension at least 20 years of age who intended to use fimasartan, and agreed to participate in the study and to be in fasting state at each visit, were included. Patients who were treated with fimasartan at baseline and premenopausal women not practicing an effective method of birth control before entry and throughout the study were excluded. Among enrolled patients, 1373 women who answered a baseline menopausal question, who were not taking estrogen, and who completed 3-month treatment with fimasartan as a first antihypertensive drug and follow-up visit were included in this study. Daily dosage of fimasartan 30, 60, or 120 mg was prescribed at the discretion of the attending physician. Postmenopausal women (post-MPW) were identified if their final menstrual period had occurred more than 1 year before the interview. Others were considered as premenopausal women (pre-MPW).

### Measurements of BP

The Omron HEM-7220 and the Omron HEM-7200 (both Omron, Tokyo, Japan) were used to measure BP in the clinic and at home, respectively. They were automated upper-arm cuff devices based on the cuff oscillometric principle. Clinic BP was measured under standardized conditions (in the same arm by the same physician or nurse). The study participants were educated about self-measurement of their own blood pressure at home. They were instructed to take their blood pressure twice in the morning and evening. An average of 2 or more BP readings at 2-minute intervals on each occasion from the same arm was recorded for 7 consecutive days. Morning BP was measured within 1 hour of awakening, after urination, in the sitting position, after resting for 5 minutes, and before taking medications or eating. In the evening, BP was measured before going to bed, after resting for 5 minutes, and in the sitting position. An average of 6 days’ recordings from the second to the seventh day was used for the analysis.^27^ Baseline assessment including health questionnaire and BP measurements were conducted before and after the 3-month treatment with fimasartan. Home BPV, which represents day-by-day BPV, was defined as the standard deviation (SD) of home BP.

### Statistical Analysis

The baseline characteristics of the study subjects were compared between pre-MPW and post-MPW using the χ^2^ test for dichotomous variables or Student *t* test for continuous variables. Changes of clinic and home BP and SD of home BP between baseline and at 3-months follow-up visit were examined using the paired *t* test. Differences of decline of BPs and SDs between 2 groups after 3 months were compared using Student *t* test. Values were considered to be statistically significant, when *P* <0.05. All statistical analyses were performed using SPSS for window version 12 (SPSS Inc, Chicago, IL).

## RESULTS

### Baseline Characteristics

In this study group, 2.5 times more women were in postmenopausal state. They were older (60.9 ± 8.2 vs. 45.3 ± 4.6 years of pre-MPW, *P* <0.001), had lower height and body weight, but had similar body mass index and waist circumference (Table 1). Post-PMW had more diabetes mellitus and dyslipidemia. About a half of patients in both groups had metabolic syndrome by Adult Treatment Panel criteria.

**TABLE 1:**
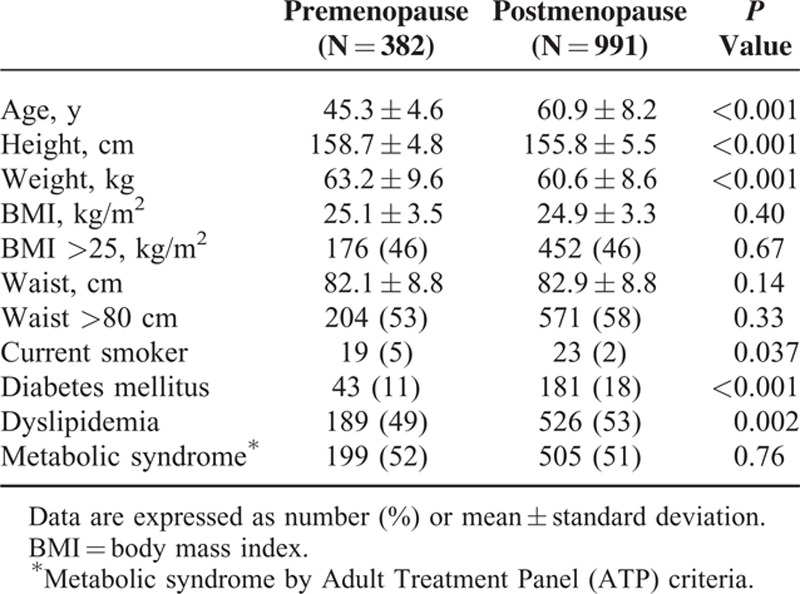
Baseline Characteristics of Patients

### Effects of Fimasartan on Clinic BP

**FIGURE 1:**
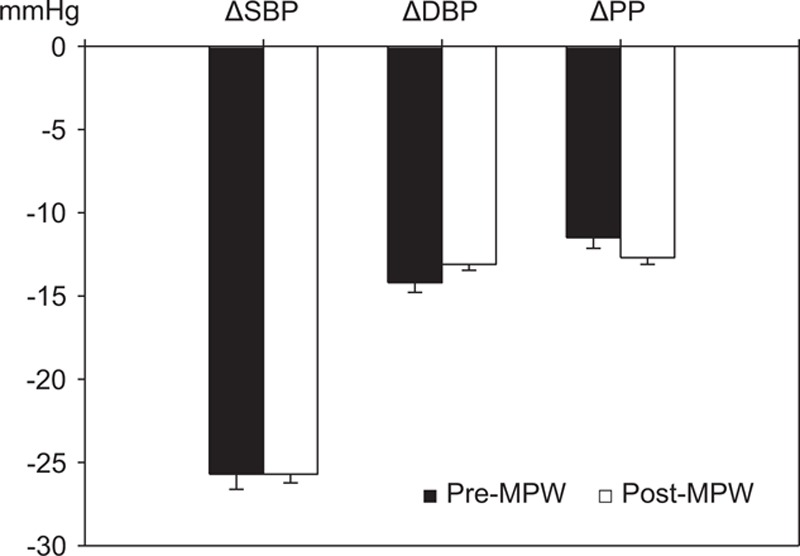
Effects of fimasartan on clinic blood pressure (BP). Fimasartan lowered clinic systolic BP (SBP), diastolic BP (DBP), and pulse pressure (PP) effectively without difference between premenopausal (pre-MPW) and postmenopausal women (post-MPW) with hypertension after 3-month treatment. Values are mean ± standard error.

**FIGURE 2:**
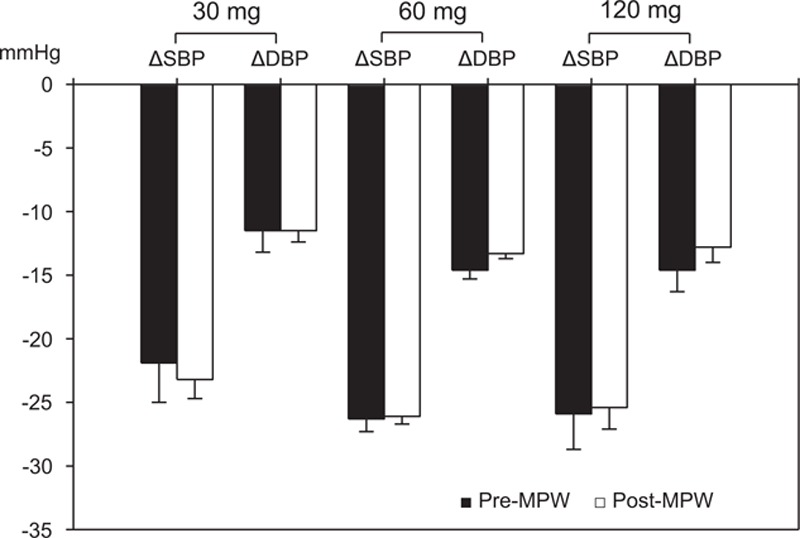
Changes of clinic blood pressure (BP) according to daily dosages of fimasartan 30, 60, or 120 mg. All dosages decreased clinic systolic BP (SBP) and diastolic BP (DBP) without difference between premenopausal (pre-MPW) and postmenopausal women (post-MPW) with hypertension after 3-month treatment. Values are mean ± standard error.

Baseline clinic systolic BP was not different between 2 groups (pre-MPW; 152.9 ± 15.2 vs. post-MPW; 152.8 ± 13.5 mm Hg, *P* = 0.89), but diastolic BP was lower (pre-MPW; 95.7 ± 9.4 vs. post-MPW; 91.9 ± 9.4 mm Hg, *P* <0.001) and pulse pressure was higher in post-MPW (pre-MPW; 57.2 ± 12.6 vs. post-MPW; 60.9 ± 12.0 mm Hg, *P* <0.001) (Table 2). Fimasartan lowered either clinic systolic or diastolic BP effectively in both groups after 3 months. Mean change of clinic systolic BP (−25.7 ± 16.3 mm Hg), diastolic BP (−13.1 ± 10.9 mm Hg), or pulse pressure (−12.7 ± 12.7 mm Hg) of post-MPW was similar to those (−25.7 ± 17.7, −14.2 ± 11.3, and −11.5 ± 12.4 mm Hg, respectively) of pre-MPW (Figure 1). Daily dosage of fimasartan was 30 mg (47 pre-MPW and 105 post-MPW), 60 mg (287 pre-MPW and 771 post-MPW), or 120 mg (48 pre-MPW and 115 post-MPW). All dosages decreased clinic systolic and diastolic BP without difference between 2 groups after 3 months (Figure 2).

**TABLE 2:**
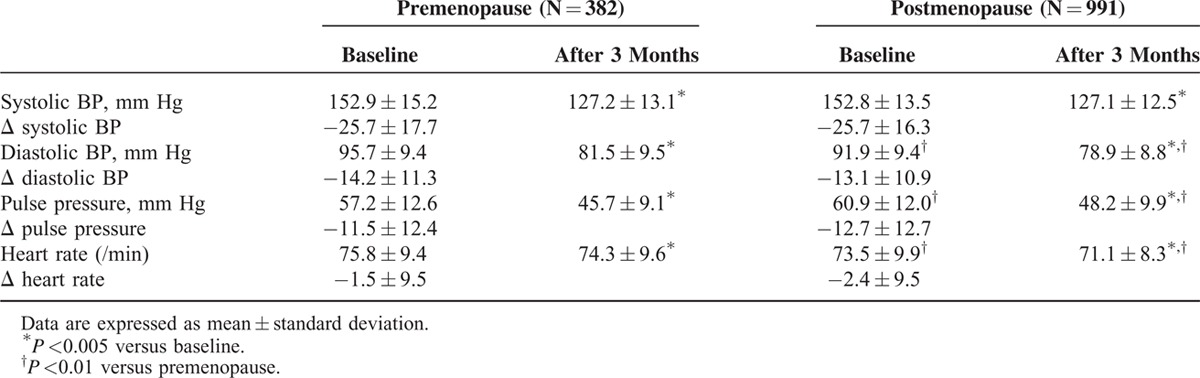
Changes of Clinic Blood Pressure (BP) After 3-Month Treatment With Fimasartan

### Effects of Fimasartan on Home BP

**FIGURE 3:**
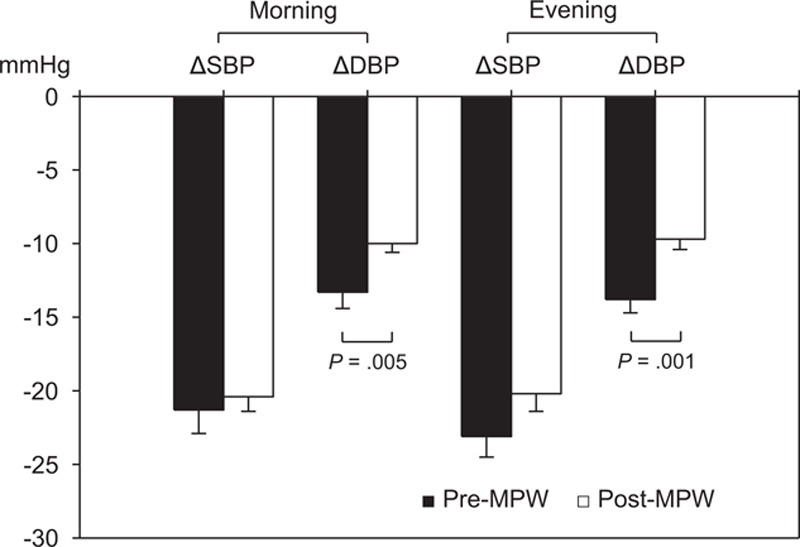
Effects of fimasartan on home blood pressure (BP). Fimasartan decreased all home morning and evening systolic BP (SBP) and diastolic BP (DBP) effectively without difference between premenopausal (pre-MPW) and postmenopausal women (post-MPW) with hypertension after 3-month treatment. Pre-MPW showed more decreased morning diastolic (*P* = 0.005) and evening diastolic BP (*P* = 0.001). Values are mean ± standard error.

Baseline morning and evening systolic BP were not different, but diastolic BP was lower and pulse pressure was higher in post-MPW (Table 3). Fimasartan decreased all home systolic BP, diastolic BP, and pulse pressure in both groups effectively after 3 months. Mean change of morning systolic BP (−20.4 ± 17.3 mm Hg) or evening systolic BP (−20.2 ± 19.2 mm Hg) of post-MPW was not different from those (−21.3 ± 17.9 and −23.1 ± 15.8 mm Hg, respectively) of pre-MPW. Morning systolic BP at 3-month was higher in post-MPW (pre-MPW; 123.1 ± 14.0 mm Hg vs. post-MPW; 127.0 ± 18.4 mm Hg, *P* = 0.031). Pre-MPW showed more decreased morning diastolic BP (pre-MPW; −13.3 ± 12.0 mm Hg vs. post-MPW; −10.0 ± 10.6 mm Hg, *P* = 0.005) and evening diastolic BP (pre-MPW; −13.8 ± 10.3 vs. post-MPW; −9.7 ± 10.9, *P* = 0.001) (Figure 3). Baseline morning and evening heart rate were greater in pre-MPW, but, after 3 months, they became similar to those of post-MPW (Table 3).

**TABLE 3:**
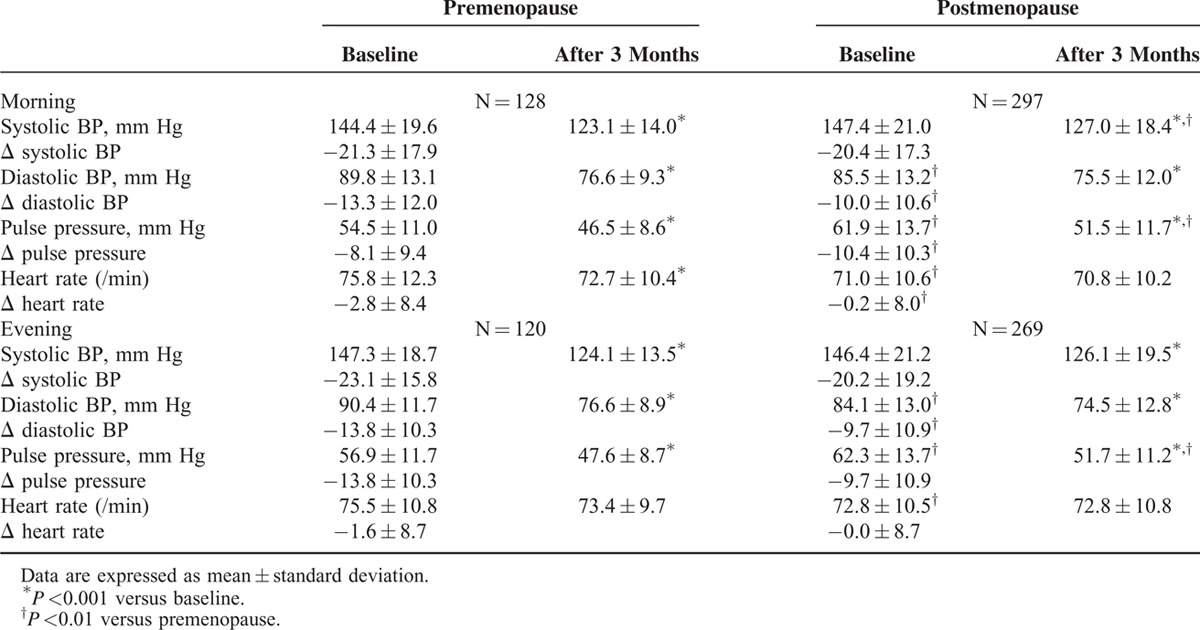
Changes of Home Blood Pressure (BP) After 3-Month Treatment With Fimasartan

Fimasartan decreased the day-to-day BPV after 3-month treatment. It lowered SDs of morning systolic BP in post-MPW (from 9.26 ± 7.31 to 7.63 ± 5.48 mm Hg, *P* = 0.001) and pre-MPW (from 8.96 ± 7.74 to 6.76 ± 5.03 mm Hg, *P* = 0.006). SDs of evening systolic BP of post-MPW (from 9.03 ± 5.44 to 7.83 ± 5.24 mm Hg, *P* = 0.002) and pre-MPW (from 9.66 ± 8.18 to 7.31 ± 5.69 mm Hg, *P* = 0.002) were also decreased after 3 months. Mean changes of SDs of morning and evening systolic BP were not significantly different between 2 groups (Table 4). SD of heart rate was not changed after 3-month treatment.

**TABLE 4:**
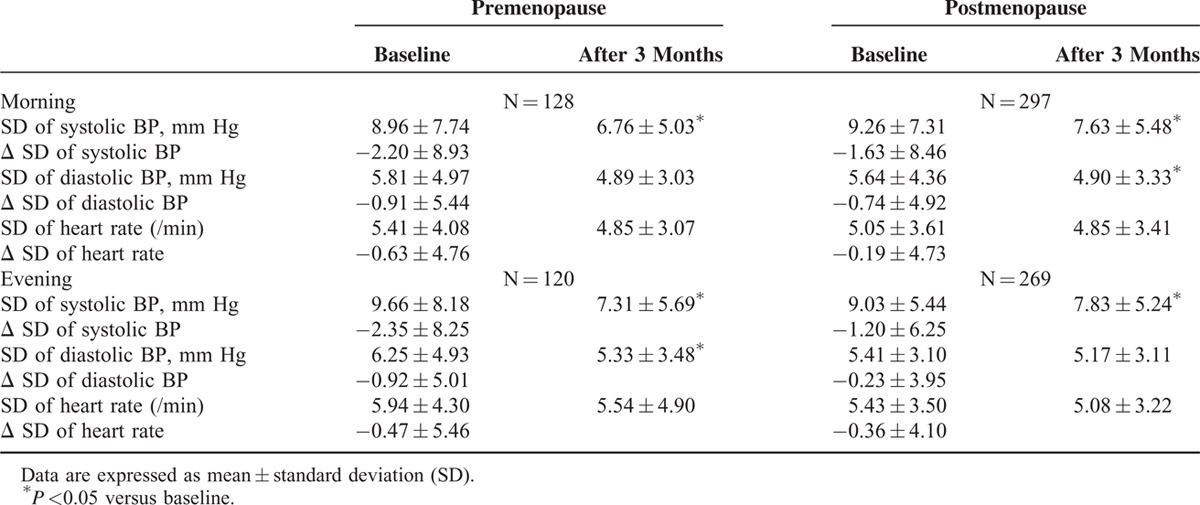
Changes of Day-to-Day Blood Pressure (BP) and Heart Rate Variability After 3-Month Treatment With Fimasartan

### Adverse Events

Adverse events were reported in 13 (3.4%) of pre-MPW and 13 (1.3%) of post-MPW (*P* = 0.015). All adverse events were minor ones such dizziness or headache except 1 case of abnormal elevation of hepatic aminotransferases in post-MPW at 3-month laboratory test. She was asymptomatic and her liver function had recovered soon after discontinuing fimasartan medication. No myocardial infarction, stroke, or death was observed during 3-month follow-up.

## DISCUSSION

This study showed that fimasartan lowered both clinic and home BP effectively in post-MPW as well as in pre-MPW with hypertension, and it also decreased day-to-day BPV.

Antihypertensive regimens based on ACEIs, ARBs, calcium antagonists, diuretics, or beta-blockers lowered BP effectively and provided protection against major CV events without difference in both genders.^28^ However, the National Health and Nutrition Examination Survey in US showed that more diuretics were prescribed in hypertensive women (32%) than in men (22%).^29^ Also in post-MPW with treated hypertension, diuretics were the most frequently used antihypertensive drug.^30^ Considering post-MPW are more salt sensitive than pre-MPW,^31^ diuretics may be a reasonable antihypertensive monotherapy. But they have several metabolic complications, and may not be an adequate choice in post-MPW with metabolic syndrome that was found in a half of post-MPW in this study. ACEIs or ARBs may be an appropriate option in this situation. In post-MPW with hypertension, an ACEI lowered diastolic BP effectively with the same amount that a diuretic did, but a diuretic had more metabolic side-effects.^18^ An ACEI showed the same BP reduction as a calcium channel blocker in postmenopausal hypertensive women.^32^ One observational cohort study of older women aged 50 to 79 years with hypertension revealed that monotherapy with calcium channel blockers was associated with 55% greater risk of CV death compared with diuretics.^33^ ACEIs had a similar CV death to diuretics, but it has been reported that women had 3 times more chance of ACEI-induced cough than men.^34^

BP-lowering effect of ARBs in post-MPW has been investigated only in a small number of patients. In a study of 51 postmenopausal women with hypertension, irbesartan efficiently decreased systolic and diastolic BP, which was potentiated by coadministration of estradiol.^35^ Both valsartan and amlodipine lowered peripheral BP and aortic pulse wave velocity without differences in a study of 125 postmenopausal women with hypertension.^19^

In this study of 991 post-MPW with hypertension, fimasartan effectively lowered both clinic systolic and diastolic BP after 3-month treatment, and mean changes of clinic systolic BP, diastolic BP, or pulse pressure of post-MPW were similar to those of 382 pre-MPW. There were also no differences between 2 groups according to daily dosage of fimasartan 30, 60, or 120 mg. Mean changes of clinic systolic/diastolic BP (−25.7 ± 16.3/−13.1 ± 10.9 mm Hg) of post-MPW were similar to those (−26.4 ± 17.3/−13.9 ± 11.6 mm Hg) reported in the naive group of Safe-KanArb study.^26^ Fimasartan also decreased home systolic BP, diastolic BP, and pulse pressure in post-MPW effectively after 3 months, and mean change of morning or evening systolic BP of post-MPW was not different from that of pre-MPW. Because baseline home diastolic BPs were higher in pre-MPW, their mean changes were greater in pre-MPW.

Old age and female sex have been known to be associated with increased home BPV.^36^ In this study, there were no significant differences of baseline morning and evening home BPV between pre-MPW and post-MPW despite post-MPW were older. Although 1 cross-sectional study in Japan showed that being medicated with ARBs was related to increased home systolic BPV in patients with antihypertensive treatment for less than 12 months,^37^ fimasartan decreased morning and evening home BPV significantly in both groups after 3 months.

Only minority of post-MPW (1.3%) complained of mild adverse events, which was less common than those of pre-MPW (3.4%). This adverse event rate was similar to 3.3% reported in Safe-KanArb study.^26^

This study has some limitations. First, menopausal status was only identified by the questionnaire, not by serum follicle-stimulating hormone level. However, several clinical and epidemiologic studies have used self-reported menstrual characteristics to define menopausal status.^19,38,39^ Second, post-MPW were older. Because age was not adjusted in statistical analysis, a few differences of baseline characteristics between pre- and post-MPW may be influenced by aging, but baseline clinic and home systolic BP was comparable between 2 groups. Third, a control group for the comparison with other ARBs or other classes of antihypertensive was not included. However, fimasartan lowered clinic and home BP at least as effectively as other kinds of antihypertensives in post-MPW with hypertension. Fourth, only 3-month BP-lowering effects of fimasartan were observed in this study. Longer-term antihypertensive difference of fimasartan between pre- and post-MPW may need to be investigated.

In conclusions, fimasartan was a similarly effective BP-lowering agent in both post-MPW and pre-MPW with hypertension, and it also decreased day-to-day BP variability.
